# Association of *CYP1A1* A4889G and T6235C polymorphisms with the risk of sporadic breast cancer in Brazilian women

**DOI:** 10.6061/clinics/2015(10)04

**Published:** 2015-10

**Authors:** Camila Borges Martins de Oliveira, Cássio Cardoso-Filho, Leonardo Silveira Bossi, Gustavo Jacob Lourenço, Maria Salete Costa-Gurgel, Carmen Silvia Passos Lima

**Affiliations:** I Universidade de Campinas, Faculdade de Ciências Médicas, Departamento de Clínica Médica, Campinas, SP, Brazil; II Universidade de Campinas, Centro de Assistência Integral à Saúde da Mulher, Campinas, SP, Brazil

**Keywords:** Sporadic Breast Cancer, *CYP1A1*, Genetic Polymorphism, Risk

## Abstract

**OBJECTIVES:**

We examined the influence of *CYP1A1* A4889G and T6235C polymorphisms on the risk of sporadic breast cancer.

**METHOD:**

DNA from 742 sporadic breast cancer patients and 742 controls was analyzed using the polymerase chain reaction, followed by the restriction fragment length polymorphism technique.

**RESULTS:**

More patients had the *CYP1A1* 4889AG+GG genotype compared to controls (29.0% *versus* 23.2%, *p*=0.004). The G allele carriers had a 1.50-fold increased risk (95% CI: 1.14–1.97) of sporadic breast cancer compared to the other study participants. The frequency of the 4889AG+GG genotype among the Caucasian patients was higher than in the non-Caucasian patients (30.4% *versus* 20.2%, *p*=0.03) and controls (30.4% *versus* 23.2%, *p*=0.002). Caucasians and G allele carriers had a 1.61-fold increased risk (95% CI: 1.20–2.15) of sporadic breast cancer compared to other subjects. The *CYP1A1* 4889AG+GG genotype was more common among patients with a younger median age at first full-term pregnancy than among controls (33.8% *versus* 23.2%, *p*=0.001) and subjects whose first full-term pregnancies occurred at an older age (33.8% *versus* 26.1%, *p*=0.03). Women with the *CYP1A1* 4889AG+GG genotype and earlier first full-term pregnancies had a 1.87-fold (95% CI: 1.32–2.67) increased risk of sporadic breast cancer compared to the other study participants. Excess *CYP1A1* 4889AG+GG (39.8% *versus* 27.1%, *p*=0.01) and 6235TC+CC (48.4% *versus* 35.9%, *p*=0.02) genotypes were also observed in patients with grade I and II tumors compared to patients with grade III tumors and controls (39.8% *versus* 23.2%, *p=*0.04; 48.4% *versus* 38.6%, *p=*0.04). The G and C allele carriers had a 2.44-fold (95% CI: 1.48–4.02) and 1.67-fold (95% CI: 1.03–2.69) increased risk, respectively, of developing grade I and II tumors compared to other subjects.

**CONCLUSIONS:**

The *CYP1A1* A4889G and T6235C polymorphisms may alter the risk of sporadic breast cancer in Brazilian women.

## INTRODUCTION

High-penetrance mutations account for a small proportion of breast cancer (BC) cases 1 and several epidemiological studies have indicated the involvement of low-penetrance genetic polymorphisms in the pathophysiology of BC 2. In addition to these genetic factors, compelling evidence supports the role of systemic hypertension, tobacco, obesity and exposure to polycyclic aromatic hydrocarbons (PAHs) in BC formation through the promotion of DNA damage and cell proliferation 3-6. Prolonged estrogen exposure is also widely accepted as a risk factor for BC due to its mitotic activity and increased mutation rates 7 and catechol estrogens can be carcinogen metabolites 8,9. Hence, some reproductive events, such as age at menarche, first-full term pregnancy (FFTP) and menopause, may play important roles in BC risk 10,11. Carriers of low-penetrance gene mutations, combined with the above-mentioned factors, might have a higher tumor risk 12,13.

The *CYP1A1* gene 14 encodes the P450-1A1 (CYP1A1) enzyme, which has aryl hydrocarbon hydroxylase activity. This enzyme converts PAHs to aryl epoxides carcinogens. The enzyme also participates in estrogen metabolism by catalyzing the 2-hydroxylation of estradiol, which results in free radical and DNA adduct production 15. Two polymorphisms in this gene have been studied in relation to BC: an A4889G substitution in exon 7 (rs1048943) and a T6235C substitution in the 3′ untranslated region (rs4646903) 16. G and C allele variants yield enzymes with increased activities in PAHs activation 17 and estrogen metabolism 18. However, the role of these variants in BC risk in individuals from the United States of America, Europe and Asia is still controversial. Some studies have reported that G and C allele variants increase BC risk 5,, while other studies have not observed main effects for *CYP1A1* variants 3,5,19-22,25,. Regarding the clinical features and biological characteristics of tumors, the impact of these genetic polymorphisms varies 3,5,10,12,15,19-23,27,. These contradictory findings might be explained by the ethnic variations among populations and small study sample sizes 41.

The Brazilian population is highly heterogeneous and consists of indigenous Amerindians and immigrants from Europe, Asia and Africa 42. Cancer is the second most common cause of death in the southeastern region of Brazil and BC represents the most common cause of tumor death among women 43. To the best of our knowledge, the role of *CYP1A1* A4889G and T6235C polymorphisms in the risk, clinical features and biological characteristics of BC tumors is unknown for people living in the Southern hemisphere. Because large numbers of participants are needed worldwide to examine the associations between a genetic polymorphism and cancer risk, we analyzed the frequencies of the *CYP1A1* A4889G and T6235C genotypes in large samples of BC patients and controls from the southeastern region of Brazil. We also investigated whether these genotypes influence the risk of BC tumors in Brazil.

## MATERIALS AND METHODS

### Study population

We analyzed 742 consecutive female patients with sporadic breast cancer (SBC) at diagnosis (median age, 53 years; range, 22–91 years; 638 Caucasians and 104 non-Caucasians). The patients were treated at the Center of Integral Attention to Women's Health of the University of Campinas, Campinas/SP, Brazil, from March 2004 to March 2007. The clinical features were determined from a questionnaire, including age at diagnosis, ethnic origin, age at menarche, age at FFTP and age at menopause, lactation status, smoking habits, oral contraceptives use and presence or absence of systemic hypertension. The patients were considered to be smokers if they kept smoking until the moment of diagnosis and the patients were classified as non-smokers if they had never smoked. The body mass index (BMI) was calculated according to the World Health Organization classification and the patients were classified into four groups: underweight, normal, pre-obese and obese 44. BC was diagnosed through the histological evaluation of tumor biopsies. The nuclear and histological grades 45 and tumor stages 46 were established according to conventional criteria. The expression levels of estrogen and progesterone receptors were analyzed through immunohistochemistry using the streptavidin-biotin complex method 47 with estrogen receptor-clone 6F11 and progesterone receptor-clone 6F16 antibodies (Novocastra Laboratories, Tyne and Wear, Newcastle, UK).

The control group was composed of 742 healthy female blood donors (median age, 40 years; range, 18–65 years; 638 Caucasians and 104 non-Caucasians) from the Centro de Hematologia e Hemoterapia of the Universidade de Campinas (during the same study period). These patients were selected to represent the general population seeking medical assistance in our region. The control group patients were matched with the BC patients according to age and ethnicity. Patients and controls with evidence of a personal or family history of BC and patients who chose not to participate in the study were excluded from the analyses. All procedures were performed according to the Declaration of Helsinki and all subjects provided written informed consent. The study was approved by the local ethics committee (process number 581/2002).

### Polymorphism analysis

The *CYP1A1* A4889G (rs1048943) and T6235C (rs4646903) genotypes were identified in genomic DNA from the peripheral blood samples of subjects using the polymerase chain reaction followed by restriction fragment length polymorphism, as previously reported 48.

### Statistical analyses

The Hardy-Weinberg (HW) equilibrium was tested using chi-squared (χ2) statistics for the goodness-of-fit (one degree of freedom). The pairwise linkage disequilibrium (LD) analysis was performed using Haploview 4.2 software (www.broad.mit.edu/mpg/haploview) to ensure that the markers were appropriate for inclusion in the haplotype estimates. The LD was measured using the disequilibrium coefficient (D′´) and strong LD significance was considered as D′´≥ 80%. Between-group differences were analyzed with χ^2^ or Fisher exact tests. A multivariate analysis using the logistic regression model was used to obtain the age and ethnic origin adjusted crude odds ratios (ORs) with 95% confidence intervals (CI) and to assess the associations between the genotypes and BC. A power of analysis (PA) was used to calculate the minimum effect size likely to be detected in a study using a given sample size, according to Pocock 49 and Hulley et al. 50 in analyses involving patients and controls and according to the DSS Research statistical power calculators 51 when considering the patients stratified by clinical and tumor characteristics. The data analyses were performed with SPSS 15.0 software (SPSS Incorporation, Chicago, IL, USA). For all statistical tests, the level of significance was 2-sided at *p*<0.05.

## RESULTS

The patient and control samples were in HW equilibrium at the *CYP1A1* A4889G (χ^2^=0.15, *p*=0.70; χ^2^=1.15, *p*=0.28) and T6235C (χ^2^=2.65, *p*=0.10; χ^2^=1.93, *p*=0.16) loci, respectively. The LD analyses revealed a moderate LD between the A4889G and T6235C polymorphisms of *CYP1A1* (D′=73%), and the referred polymorphisms were considered for further analysis of the haplotypes.

More patients had the *CYP1A1* 4889AG (PA=87.0%) and *CYP1A1* 4889AG+GG (PA=93.0%) genotypes compared with the controls. Compared with the other study participants, carriers of the*CYP1A1* 4889AG genotype had a 1.45-fold increased risk of SBC and carriers of the *CYP1A1* 4889AG+GG genotypes had a 1.50-fold increased risk.</emph> Similar frequencies of the *CYP1A1* T6235C genotypes and *CYP1A1* haplotypes were observed in the patients and controls enrolled in the study (Table 1).

The *CYP1A1* 4889AG and 4889AG+GG genotypes were more common in the Caucasian SBC patients than in the non-Caucasian patients (PA=73.0% and PA=57.3%, respectively) and controls (27.9% *versus* 21.1%, *p*=0.002, PA=98% and 30.4% *versus* 23.2%, *p*=0.002, PA=98%, respectively). Caucasians with the *CYP1A1* 4889AG and 4889AG+GG genotypes had a 1.61-fold and 1.61-fold increased risk of tumors, respectively, than Caucasians with the remaining genotypes, respectively. An excess of the *CYP1A1* 6235CC genotype was observed in SBC patients with a lower median age of menarche (younger than 13 years of age) compared to patients who experienced menarche at a higher median age (13 years of age and older, PA=55.3%). The *CYP1A1* 4889AG+GG genotype was more common among patients with a lower median age at FFTP (younger than 22 years of age) than among patients who experienced FFTP at a higher median age (22 years of age and older) PA=58.6%). The frequency of the *CYP1A1* 4889AG+GG genotype was higher among patients with an earlier FFTP than among the controls (33.8% *versus* 23.2%, *p=*0.001, PA>99.0%). Women with the *CYP1A1* 4889AG+GG genotype and FFTP occurring earlier in life had a 1.87-fold increased risk of SBC compared to subjects with the AA genotype. The frequency of the *CYP1A1* 6235CC genotype in pre-obese and obese patients was higher than in normal and underweight patients (PA=32.1%). Moreover, the *CYP1A1* 4889AG (PA=77.1%), *CYP1A1* 4889AG+GG (PA=69.3%) and *CYP1A1* 6235TC+CC (PA=63.0%) genotypes were more common among patients with I and II histological grade tumors than among patients with grade III tumors. The frequencies of the respective genotypes in patients from the first group were also higher than those observed in the controls (37.6% *versus* 21.2%, *p*<0.001, PA>99.0%; 39.8% *versus* 23.2%, *p<*0.001, PA>99.0%; 48.4% *versus* 38.6%, *p=*0.04, PA=95.0%). Individuals with the *CYP1A1* 4889AG, *CYP1A1*AG+GG, and *CYP1A1* 6235TC+CC genotypes had 2.48-, 2.44- and 1.67-fold increased risks, respectively, for SBC compared to study participants with the remaining genotypes (Table 2). Similar frequencies of the *CYP1A1* A4889G and T6235C genotypes were observed among patients stratified by age at diagnosis, age at menarche (younger than 12 years *versus* 12 years and older), age at menopause and FFTP (younger than 30 years *versus* 30 years and older), oral contraceptive use, lactation, cigarette smoking habit, systemic hypertension, tumor histology, stage and estrogen and progesterone receptor patterns. The frequencies of the *CYP1A1* haplotypes did not differ among patients stratified by clinical and tumor features (data not shown).

## DISCUSSION

In this case-control study, we examined the influence of the *CYP1A1* A4889G and T6235C polymorphisms on the risk of SBC. We also investigated whether these polymorphisms are associated with the clinical features of SBC patients and with the biological tumor characteristics.

We initially found that the *CYP1A1* 4889AG and 4889AG+GG genotypes were associated with a higher risk of BC in our cohort. Our results were consistent with the findings of Chacko et al. 23, Singh et al. 36 and Surekha et al. 27 (studies conducted in India). Ambrosone et al. 19 and Ishibe et al. 5 also reported an increased risk of BC among G variant allele carriers (with smoking habits) from the United States of America. In fact, cytochrome P450 enzymes encoded by the *CYP1A1* gene play an important role in the phase I bioactivation of xenobiotics and in the metabolism of estrogen (converting the metabolites into carcinogens) 15. G variant allele carriers of the *CYP1A1* A4889G polymorphism have a higher catalytic enzyme activity 17,18, which results in a higher level of carcinogens in breast tissue and, consequently, a higher level of DNA adducts, which may serve as the initial step in BC development. In contrast with our study, the *CYP1A1* 4889AG genotype was associated with a lower risk of BC among Japanese 40 patients. Furthermore, no association between the *CYP1A1* 4889AG polymorphism and BC risk was observed among North Americans 19-22,30,33,, Chinese 21,34, Europeans 31, or Indians 25,28. These discrepant results seem to be due to ethnic variations among the studied populations because the *CYP1A1* 4889AG+GG genotype was both underrepresented 21,25,28,34,40 and overrepresented 19,20,22,30,31,33,35 in our controls compared with controls from the other studies.

In our study, the *CYP1A1* T6235C polymorphism was not associated with SBC risk. Although a large number of subjects were evaluated, the statistical analyses were characterized by a low statistical power. In addition, the *CYP1A1* T6235C polymorphism did not alter the risk of BC among African-Americans 30,33, North-Americans 5,21,30,33, Chinese 34, Indians 25,36, or Brazilians 37. In contrast, Miyoshi et al. 40 and da Fonte de Amorim et al. 32 reported a lower risk of BC among Japanese and Brazilian carriers of the C variant allele. Moreover, an association between the *CYP1A1* T6235 C allele and a high BC risk was previously described by Taioli et al. 20,22, Li et al. 33, Chacko et al. 23, Shen et al. 24, Gulyaeva et al. 26, Naushad et al. 28 and Khvostova et al. 29. Discrepancies between the results of our study and the da Fonte de Amorim study 32 (both studies were conducted among Brazilians) and discrepancies between the results of our study and those of Taioli 20,22 and Naushad 28 (African-American and Indian women, respectively) may be attributed to different sample sizes. We analyzed 742 SBC patients. The studies by da Fonte de Amorim, Taioli and Naushad analyzed significantly smaller numbers of patients (n=128, n=57 and n=342, respectively). Conversely, the discrepancies between our study and the remaining discordant studies may be explained by ethnic variation within the populations because the frequency of the *CYP1A1* 6235TC+CC genotype was lower 40 or higher 23,24,26,29,33 among our controls compared to their controls.

Additionally, in our study, the *CYP1A1* 4889AG and 4889AG+GG genotypes were overrepresented in Caucasian Brazilian women with BC compared with non-Caucasian women. The frequencies of the referred genotypes were also higher among our Caucasian SBC patients than in our control group. Therefore, Caucasian women carrying the G allele had a higher risk of SBC development. Our finding is consistent with the Sergentanis & Economopoulos meta-analysis 52, which included Caucasian, Chinese and African populations from 29 eligible studies. We also observed similar frequencies of the distinct *CYP1A1* T6235C polymorphisms among our Caucasian and non-Caucasian women. To the best of our knowledge, the association between the *CYP1A1* T6235 C allele and a high risk BC was only observed in a small study of North-Americans, which was conducted by Taioli et al. 20,22.

We identified a relationship between the *CYP1A1* 6235CC genotype and SBC in women who experienced an earlier age at menarche compared to women who experienced a later age at menarche, as previously described by Huang et al. 21,39. In fact, women with early menarche are exposed to estrogen for a longer period of time, which might amplify the effect of the *CYP1A1* 6235CC variant product on breast tissue. We also surprisingly found that the *CYP1A1* 4889AG+GG genotype was overrepresented in SBC patients who experienced their FFTP earlier in life compared to women who experienced a later FFTP. An early age at FFTP is well-known to be highly protective against the onset of BC 11,53,54 and the *CYP1A1* A4889G genotype is associated with an increased BC risk in women with an older age at FFTP 55,56 because of the prolonged estrogen exposure of the breast epithelium 6,10. However, Brazilian women are characterized by a low average FFTP age and also by multiparity 57, which seem to be protective factors 11. However, Kobayashi et al. 58 reported that each pregnancy, including the first one, increases the risk of early onset BC due to the reaction cascade triggered by chorionic gonadotropin hormone (hCG) during the first trimester of pregnancy. This cascade results in the morphological and functional development of breast tissue. The high progesterone conditions induced by hCG seem to be essential to pregnancy-associated breast development, presumably as a result of mammary epithelial stem cell expansion. This fact, associated with the high estrogen metabolism among multiparous women with the G variant allele, may explain our results. However, we did not determine how many pregnancies each woman had experienced until BC was diagnosed.

In our study and Huang et al. 21,39, BC risk was higher among women with a high BMI. In Huang et al., the CC variant genotype of the *CYP1A1* T6235C polymorphism was overrepresented in pre-obese and obese women, suggesting that the CC variant might affect BC susceptibility. One plausible mechanism for this association is that obese women have a higher circulating estrogen level caused by the conversion of androgen to estrogen in adipose tissue. Apart from this possibility, obese women have a higher formation rate of genotoxic compounds that may lead to DNA adduct formation in mammary cells 10,11.

Finally, we observed that the G and C variant alleles of both polymorphisms were positively associated with lower histological grade tumors, thereby indicating their roles in the progression of mammary carcinogenesis (even compared to controls). Thus, carriers of the respective variant alleles of both polymorphisms have an increased risk of developing histological grade I and II BC. Conflicting results regarding the roles of *CYP1A1* A4889G and T6235C in the histological grade of BC have been previously observed in small studies conducted by Miyoshi et al. 40 (n=195) and Singh et al. 36 (n=150).

Our data are useful for predicting BC risk. Our results showed that *CYP1A1* A4889G and T6235C polymorphisms alter the risk and clinical or tumor characteristics of BC among a heterogeneous population from Southeastern Brazil. Although this study is not the first study to investigate SBC in Brazil 32,37, the large sample size provides more reliable conclusions regarding SBC among Brazilians.

## Figures and Tables

**Table 1:** *CYP1A1* genotypes among sporadic breast cancer patients and controls.

Genotypes	Patients Number (%)	Controls Number (%)	*p-*value	OR* (95% CI^a^)
**A4889G**				
AA	527 (71.0)	570 (76.8)		reference
AG	195 (26.3)	157 (21.2)	**0.01**	**1.45 (1.10-1.93)**
GG	20 (2.7)	15 (2.0)	0.09	2.05 (0.89-4.71)
AA	527 (71.0)	570 (76.8)		reference
AG+GG	215 (29.0)	172 (23.2)	**0.004**	**1.50 (1.14-1.97)**
AA+AG	722 (97.3)	727 (98.0)		reference
GG	20 (2.7)	15 (2.0)	0.14	1.84 (0.81-4.20)
**T6235C**				
TT	464 (62.5)	456 (61.5)		reference
TC	236 (31.8)	243 (32.7)	1.00	1.00 (0.77-1.30)
CC	42 (5.7)	43 (5.8)	0.74	1.09 (0.65-1.83)
TT	464 (62.5)	456 (61.4)		reference
TC+CC	278 (37.5)	286 (38.6)	0.90	1.01 (0.79-1.30)
TT+TC	700 (94.3)	699 (94.2)		reference
CC	42(5.7)	43 (5.8)	0.70	1.10 (0.66-1.83)
**A4889G/T6235C**				
AA+TT	413 (74.5)	438 (76.3)		reference
AG+TC	128 (23.1)	123 (21.4)	0.18	1.25 (0.90-1.74)
GG+CC	13 (2.4)	13 (2.3)	0.34	1.59 (0.61-4.15)
AA+TT	413 (71.6)	438 (74.0)		reference
AG+GG+TC+CC	164 (28.4)	154 (26.0)	0.14	1.26 (0.93-1.70)
AA+AG+TT+TC	693 (98.1)	697 (98.2)		reference
GG+CC	13 (1.9)	13 (1.8)	0.44	1.45 (0.56-3.74)

**<?ENTCHAR ast?>:** : Adjusted odds ratio for age and ethnic origin; **^a^**: confidence interval. The significant results are shown in a bold font.

**Table 2:** . *CYP1A1* genotypes among sporadic breast cancer patients stratified by the clinical features and biological aspects of the tumor

		*CYP1A1* A4889G polymorphism						
Features	No^a^	AA	AG	GG	AA+AG	GG	AA	AG+GG
**Ethnical origin**	742							
Caucasian	638	444 (69.6)	**178 (27.9)**	16 (2.5)	622 (97.5)	16 (2.5)	**444 (69.6)**	**194 (30.4)**
Non-Caucasian	104	83 (79.8)	**17 (16.3)**	4 (3.9)	100 (96.1)	4 (3.9)	**83 (79.8)**	**21 (20.2)**
*p-*value			**0.01**	0.64	0.47	</emph>	**0.03**	
**MAM^b^**	693**^c^**							
younger than 13 years	298	202 (67.8)	89 (29.9)	7 (2.3)	291 (97.7)	7 (2.3)	202 (67.8)	96 (32.2)
13 years and older	395	289 (73.1)	93 (23.5)	13 (3.4)	382 (96.6)	13 (3.4)	289 (73.1)	106 (26.9)
*p-*value			0.12	0.58	0.48		0.19	
**MAFFTP^d^**	692**^c^**							
younger than 22 years	275	182 (66.2)	82 (29.8)	11 (4.0)	264 (96.0)	11 (4.0)	**182 (66.2)**	**93 (33.8)**
22 years and older	417	306 (73.4)	103 (24.7)	8 (1.9)	409 (98.1)	8 (1.9)	**306 (73.4)**	**111 (26.1)**
*p-*value			0.08	0.08	0.11		**0.03**	
**Body mass index**	708^c^							
Under+normal	253	185 (73.1)	63 (24.9)	5 (2.0)	248 (98.0)	5 (2.0)	185 (73.1)	68 (26.9)
Pre-obese+obese	455	316 (69.5)	125 (27.5)	14 (3.0)	441 (97.0)	14 (3.0)	306 (69.5)	139 (30.5)
*p-*value			0.29	0.29	0.33		0.21	
**Histological grade**	642**^c^**							
I+II	93	**56 (60.2)**	**35 (37.6)**	2 (2.2)	91 (97.8)	2 (2.2)	**56 (60.2)**	**37 (39.8)**
III	549	**400 (72.9)**	**132 (24.0)**	17 (3.1)	532 (96.9)	17 (3.1)	**400 (72.9)**	**149 (27.1)**
*p-*value			**0.008**	0.83	0.62		**0.01**	

		*CYP1A1* T6235C polymorphism						
Features	No^a^	TT	TC	CC	TT+TC	CC	TT	TC+CC
**Ethnical origin**	742							
Caucasian	638	403 (63.2)	202 (31.7)	33 (5.1)	605 (94.9)	33 (5.1)	403 (63.2)	235 (36.8)
Non-Caucasian	104	61 (58.7)	34 (32.7)	9 (8.6)	95 (91.4)	9 (8.6)	61 (58.7)	43 (41.3)
*p-*value			0.70	0.21	0.20		0.45	
**MAM^b^**	693**^c^**							
younger than 13 years	298	181 (60.7)	94 (31.6)	**23 (7.7)**	275 (92.3)	23 (7.7)	181 (60.7)	117 (39.3)
13 years and older	395	255 (64.6)	124 (31.4)	**16 (4.0)**	379 (96.0)	16 (4.0)	255 (64.6)	140 (35.4)
*p-*value			0.78	**0.04**	0.05		0.37	
**MAFFTP^d^**	692**^c^**							
younger than 22 years	275	165 (60.0)	94 (34.1)	16 (5.9)	259 (94.1)	16 (5.9)	165 (60.0)	110 (40.0)
22 years and older	417	263 (63.1)	129 (30.9)	25 (6.0)	392 (94.0)	25 (6.0)	263 (63.1)	154 (36.9)
*p-*value			0.39	0.93	0.87		0.44	
**Body mass index**	708^c^							
Under+normal	253	159 (62.8)	84 (33.2)	10 (4.0)	**243 (96.0)**	**10 (4.0)**	159 (62.8)	94 (37.2)
Pre-obese+obese	455	287 (63.1)	137 (30.1)	31 (6.8)	**424 (93.2)**	**31 (6.8)**	287 (63.1)	168 (36.9)
*p-*value			0.72	0.05	**0.04**		0.76	
**Histological grade**	642**^c^**							
I+II	93	48 (51.6)	37 (39.8)	8 (8.6)	85 (91.4)	8 (8.6)	**48 (51.6)**	**45 (48.4)**
III	549	352 (64.1)	169 (30.8)	28 (5.1)	521 (94.9)	28 (5.1)	**352 (64.1)**	**197 (35.9)**
*p-*value			0.05	0.07	0.18		**0.02**	

a : number; **^b^**: Median age at menarche; **^c^**: The total number differs from the total (742) number quoted in the study because it was not possible to obtain information for some individuals; **^d^**: Median age at first full-term pregnancy. The significant results are presented in a bold font.
